# Mycobacterium bovis Bacillus Calmette-Guérin (BCG)-Related Sternal Osteomyelitis

**DOI:** 10.7759/cureus.78054

**Published:** 2025-01-27

**Authors:** Belkis Hatice Inceli, Döndü Nilay Penezoglu, Halil Özdemir, Gül Arga, Seda Kaynak Sahap, Ömer Suat Fitoz, Koray Ceyhan, Ayten Kayi Cangir, Zehra Sule Haskologlu, Ergin Çiftçi

**Affiliations:** 1 Pediatric Infectious Diseases, Ankara University School of Medicine, Ankara, TUR; 2 Pediatric Radiology, Ankara University School of Medicine, Ankara, TUR; 3 Pathology, Ankara University School of Medicine, Ankara, TUR; 4 Thoracic Surgery, Ankara University School of Medicine, Ankara, TUR; 5 Pediatric Allergy and Immunology, Ankara University School of Medicine, Ankara, TUR

**Keywords:** bcg, granulomatous inflammation, mycobacterium bovis, osteomyelitis, sternum

## Abstract

Although the Bacillus Calmette-Guérin (BCG) vaccine causes some complications such as lymphadenitis, cellulitis, and localized musculoskeletal diseases after administration, post-vaccine osteomyelitis is one of the rare complications in healthy children. Diagnosis is often delayed due to atypical presentation. An 11-month-old boy with a normal immune system, who was previously known to be completely healthy and had been vaccinated with BCG, was referred to our center due to a newly noticed firm swelling on the anterior chest wall. There was no evidence of previous tuberculosis infection or contact with a tuberculosis patient. In thorax computed tomography (CT) and magnetic resonance imaging (MRI), there was an irregular soft tissue appearance with heterogeneous contrast, causing lytic changes in the sternum. Histopathological examination of the fine needle aspiration biopsy performed for diagnostic purposes was found to be compatible with post-vaccination BCG-related granulomatous osteomyelitis. Mycobacterium bovis was isolated in the culture sent from the biopsy material. The patient was started on isoniazid, rifampicin, ethambutol, and ciprofloxacin treatments. No complications developed during the treatment, and in the follow-up imaging one month later, a significant shrinkage of the lesion (90%) was detected compared to the previous examination.

## Introduction

The Bacillus Calmette-Guérin (BCG) vaccine is a Mycobacterium bovis strain that has all the structural features of tuberculosis bacillus, but whose disease-causing potential was eliminated. The vaccine has been administered intradermally since 1945 in many countries of the world for the development of an active immune response against tuberculosis and to prevent the disease [1]. The effectiveness of the vaccine in preventing miliary tuberculosis and tuberculous meningitis is well-known [2]. The vaccine has a high safety profile in children with normal immune systems while its application in immunocompromised children may cause life-threatening complications [1,2]. Post-vaccination complications may also occur in children having normal immunity and the most common ones are abscesses limited to the vaccination site and regional suppurative lymphadenitis [3,4]. While regional suppurative lymphadenitis is generally seen 3 to 28 weeks after vaccination, systemic complications, such as pulmonary tuberculosis and cervical adenitis (scrofula), meningitis, and osteomyelitis, are seen 1 month to 3 years later [5,6]. One of the rare complications of the BCG vaccine is osteomyelitis. Long bone epiphyses are frequently affected, but osteomyelitis cases involving flat bones, such as the sternum, are reported, though rarely [7-10]. In this study, a case of sternal osteomyelitis developed after BCG vaccination in an 11-month-old boy who was known to be completely healthy and had an apparently normal immune system.

## Case presentation

**Figure 1:**
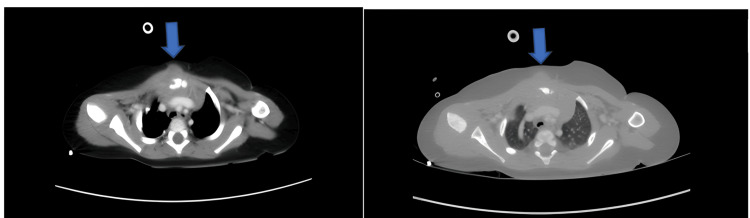
Thoracic CT image of the patient taken in our hospital

An 11-month-old male patient, who was previously known to be healthy, presented after his mother discovered a firm swelling on the anterior chest wall while she was bathing him 10 days before his admission to our hospital. The family applied to the child health and diseases clinic, and after the initial evaluation, they were referred to the pediatric oncology department. The patient underwent a thoracic CT and MRI. The mass, which involved the right half of the sternum, the first costochondral joint, and extended to the anterior mediastinum, deep to the right pectoral muscle, and the subcutaneous tissue in the midline, was reported as chondrosarcoma (Figure 1).

**Figure 2:**
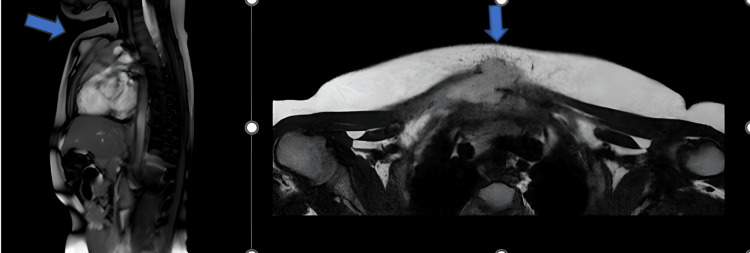
Appearance of a mass extending to the subcutaneous tissue in the midline of the retrosternal region in the thoracic MRI taken at the patient's first admission

The family voluntarily applied to our hospital's thoracic surgery first and then to the pediatric oncology department. It was planned to perform controlled thoracic imaging and take a biopsy of the lesion. In the radiological evaluation of the thoracic CT taken in our hospital, irregular soft tissue appearance with heterogeneous contrast, causing lytic change and increased sclerosis in the right manubrium-sternal half, extending to the retrosternal region and skin, and millimetric non-enhancing areas that may belong to necrosis were observed. The obtained appearance was stated to be compatible with osteitis/osteomyelitis and a biopsy was recommended to exclude malignancy (Figure 2).

The pathology result of the ultrasound-guided fine-needle aspiration by interventional radiology revealed findings consistent with necrotizing granulomatous inflammation, but it was stated that no further interpretation could be made due to insufficient biopsy material. The patient was hospitalized at our Pediatric Infectious Diseases Department to be examined for osteomyelitis and granulomatous inflammation and to make a treatment plan. The patient had no known history of disease, trauma, or surgery. He had no symptoms such as fever, cough, weight loss, or night sweats. There was no family history of active tuberculosis. Moreover, the patient had no history of contact with anyone suffering from tuberculosis.

On physical examination, the body temperature was 36.5 °C, blood pressure was 90/50 mmHg, and pulse rate was 110/minute; growth and development and especially weight status were normal. During the system examination, a swelling on the sternum, raised from the skin and not covered with redness, was observed. On palpation, a hard, motionless, and painless mass with a diameter of 2 cm was felt. Other system examinations were normal.

No abnormalities were detected in the complete blood count and biochemistry. His serology for HIV, HCV, and HBV was negative. No parenchymal infiltration or mediastinal width was observed on two-way chest radiography. The patient was started on ampicillin-sulbactam and teicoplanin treatments due to suspicion of osteomyelitis. A tuberculin skin test was performed on the patient, who had been vaccinated in accordance with the months (BCG vaccine was administered at two months of age) and had a BCG scar, and the induration diameter was measured as 12 mm. A three-day fasting gastric juice was taken from the patient to check for tuberculosis. It was sent for acid-fast bacilli (AFB), polymerase chain reaction (PCR) examination, and culture, which resulted as negative. No pathology was detected in the screening performed on the family for tuberculosis.

Pediatric rheumatology was consulted for rheumatological diseases that could cause granulomatous inflammation. It was stated that the patient was not primarily considered to have vasculitis, and it was recommended to send angiotensin-converting enzyme (ACE), perinuclear antineutrophil cytoplasmic antibody (p-ANCA), cytoplasmic antineutrophil cytoplasmic antibody (c-ANCA) panels and to perform eye examination. The ACE level was normal, p-ANCA and c-ANCA results were negative, and no pathology was detected in the eye examination. The patient's pediatric immunology evaluation was also performed for immunodeficiency and immunoglobulins, vaccine responses, T-B lymphocyte functions, and CD212 values were normal. He was investigated for chronic granulomatous disease; the examinations were normal too. Genetic screening was performed and no genetic disorder was detected.

**Figure 3:**
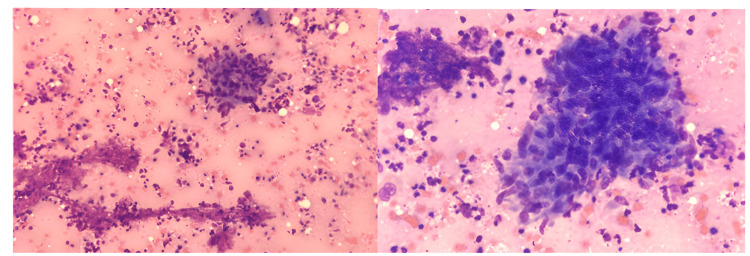
Epithelioid histiocyte aggregate and necroinflammatory background, characteristic of necrotizing granuloma, are seen in this cytological smear (May-Grünwald-Giemsa stain)

**Figure 4:**
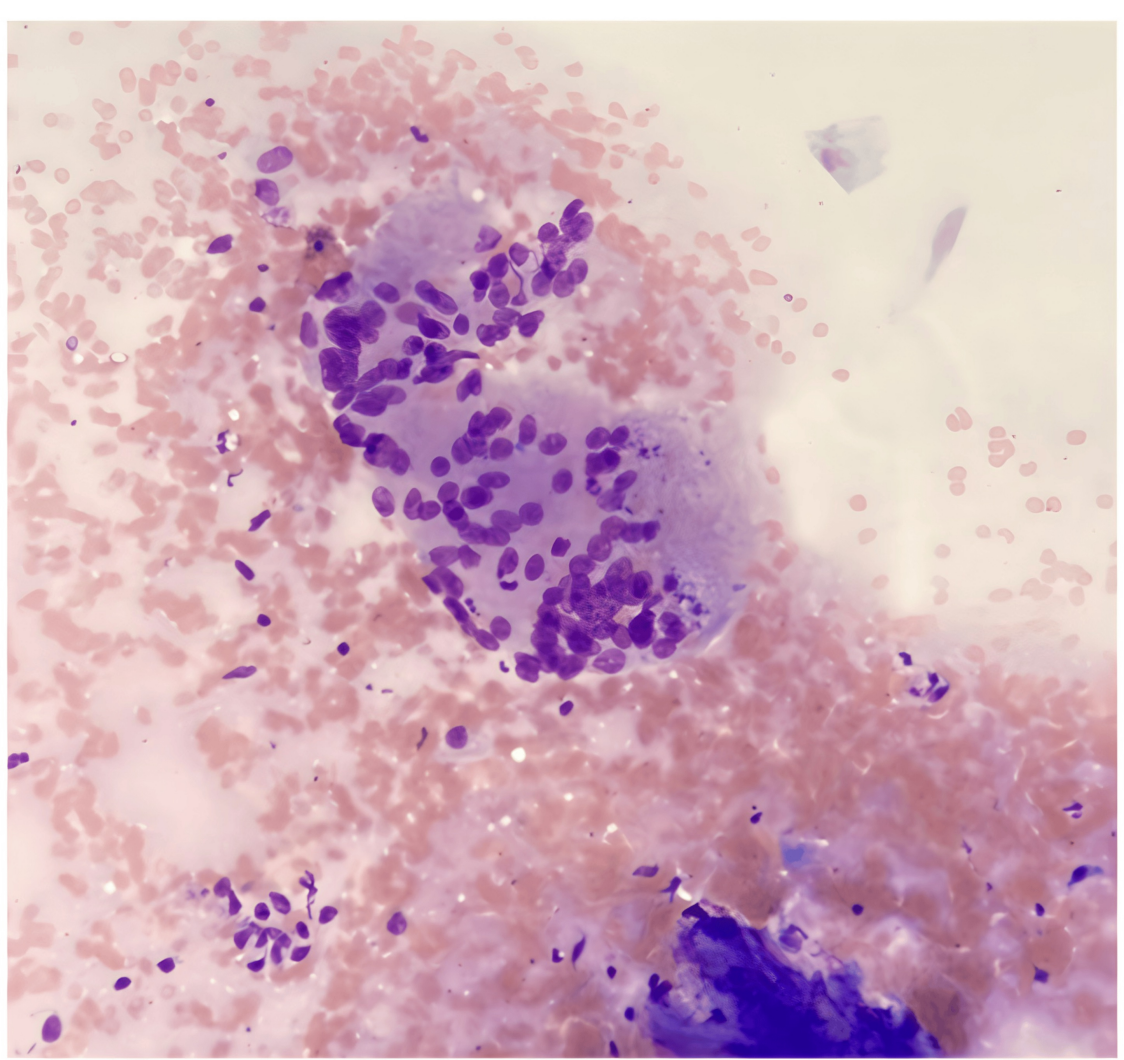
A multinucleated histiocytic giant cell (Langhans cell), another salient cytological feature of granulomatous reaction is observed (May-Grünwald-Giemsa stain)

**Figure 5:**
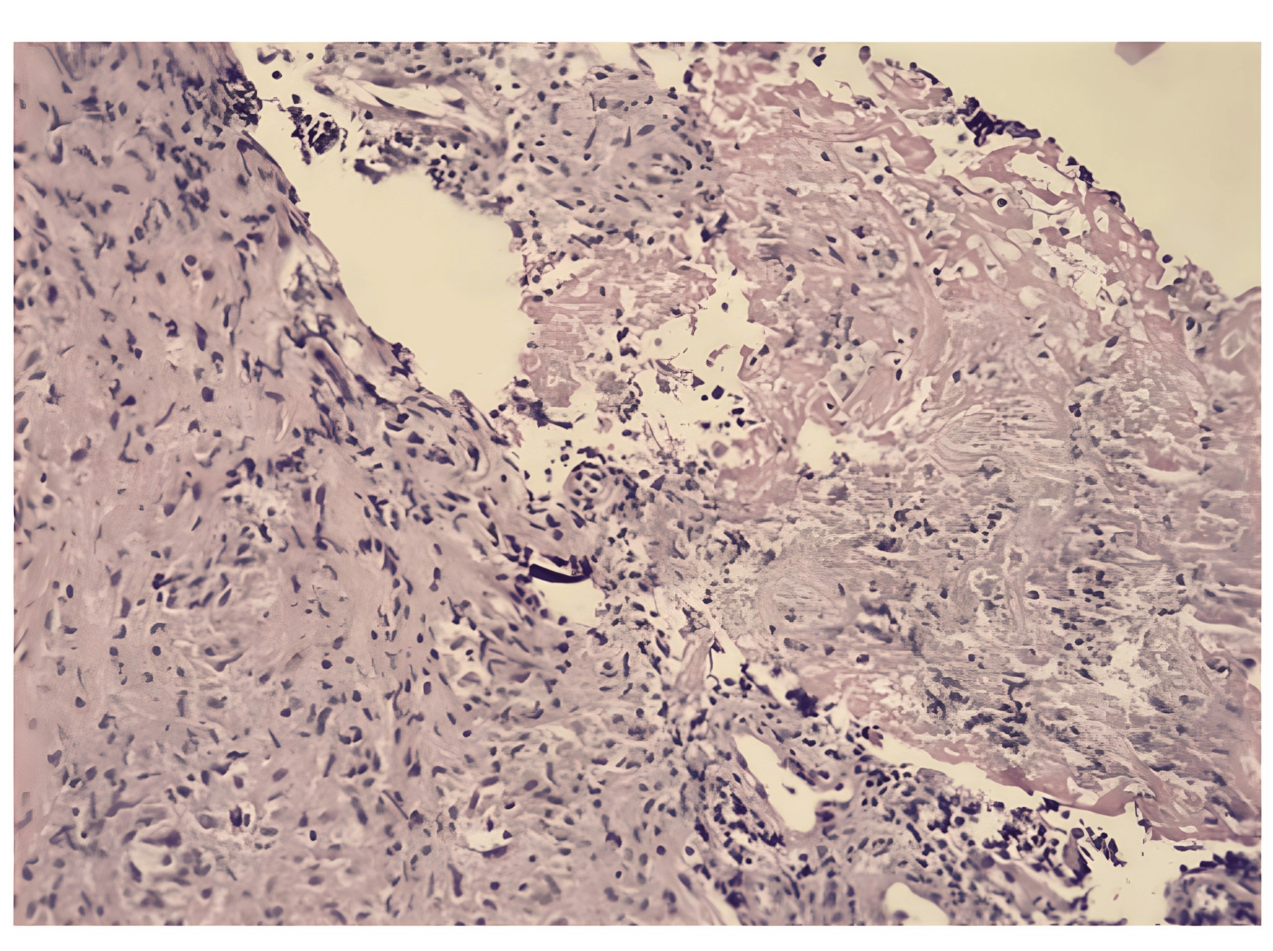
Fine-needle aspiration-derived cell block section of the lesion shows central caseous necrosis and surrounding granulomatous reaction including epithelioid histiocytes and lymphocytes (Hematoxylin-Eosin stain)

The patient underwent another fine-needle aspiration biopsy in the interventional radiology department, with the consent of the family. As a result of the pathology, it was stated that multinucleated histiocytic giant cells and epithelioid histiocytes suggesting granulomatous inflammation were observed, and post-vaccination BCG-related granulomatous osteomyelitis was primarily considered (Figures 3-5). The ARB staining and TBC PCR were negative for the tissue. In light of these findings, it was decided to start quadruple anti-tuberculosis treatment for the patient, consisting of isoniazid (10 mg/kg/dose, single dose, oral), rifampicin (15 mg/kg/dose, single dose, oral), ethambutol (20 mg/kg/dose, single dose, oral), and ciprofloxacin (10 mg/kg/dose, 2 doses, oral). The ampicillin-sulbactam and teicoplanin treatments he was receiving were discontinued after completing 10 days.

Mycobacterium tuberculosis complex growth was detected in the mycobacteria culture sent from the patient's biopsy material and studied with the BACTEC MGIT 960 system (Becton Dickinson, Franklin Lakes, NJ, US). The sample sent to the National Tuberculosis Reference Laboratory for species determination was found to be Mycobacterium bovis. The patient was able to tolerate anti-tuberculosis medications, and no problems occurred after the treatment. He was discharged with the condition that a hemogram and biochemistry would be performed two weeks later, and a thoracic CT check would be performed one month later. The abdominopelvic ultrasonography was normal. No pathology was detected in control examinations. Contrast-enhanced thorax CT revealed a significant shrinkage of the lesion (90%) compared to the previous examination. The patient's follow-up continues and the antituberculosis treatment was discontinued after one year.

## Discussion

Tuberculosis is an important public health problem related to socioeconomic level, nutrition, and general hygiene conditions. The BCG vaccine has been used to prevent tuberculosis for over 100 years and has a good safety profile [8-12]. It is generally well-tolerated. However, some serious side effects may occur after injection depending on the vaccine dose, vaccine strain, injection technique, and whether the person has an underlying disease [13,14].

Post-vaccination complications occur in 3.3% of patients and usually occur 6 to 9 months after vaccination [9]. The most common complications are localized abscesses and suppurative lymphadenitis [9-12]. Of the 92 BCG vaccine reactions reported in Japan in 2010, suppurative lymphadenitis was detected in the axillary lymph nodes in 42, cutaneous tuberculosis in 21, subcutaneous abscess in the infection area in 7, and osteomyelitis in 5.

Osteomyelitis is a very rare late complication of BCG in immunocompetent patients, with an incidence of 1 in 80,000 to 100,000 vaccinations. BCG osteomyelitis frequently affects the extremity bones and, rarely, the ribs and sternum [10-14]. In most cases, the epiphysis or metaphysis is affected. In a multicenter study conducted in Taiwan including 71 children diagnosed with BCG osteomyelitis, 36.6% of children had lower extremity long bone involvement, followed by foot bone (23.9%), rib or sternum (15.5%), upper extremity long bone (9.9%), hand bone (7%), multiple bones (4.2%), and vertebrae (2.8%). Three children with vertebral and multifocal infections had two major sequelae, such as kyphosis or leg length discrepancy. The results were better in children with rib, sternum, and peripheral bone involvement without multifocal involvement. The average duration of functional recovery was 6.2±3.9 months [15]. In some pediatric cases, tuberculosis of the short tubular bones like phalanges, metacarpals, or metatarsals is quite uncommon after the age of five years, once the epiphyseal centers are well-established. Radiographic features of cystic expansion of the short tubular bones have led to the name “spina ventosa” for tuberculous dactylitis of the short bones. Ikincioğullari et al. reported a case of tuberculous osteomyelitis presenting as hand spina ventosa, hepatosplenomegaly, and soft tissue abscess in the phalanges in a six-month-old baby who had a fully compatible bone marrow transplant for T-B+severe combined immunodeficiency (SCID) and subsequently had an unexplained fever for four weeks or more [16].

BCG osteomyelitis is more common in men. In a series of 22 cases reported by Koyama et al., the male-to-female ratio was found to be 16:5 [14]. BCG osteomyelitis is a disseminated infection. Although the BCG vaccine is administered intradermally, the microorganism spreads to the blood and the bone via blood. Redness and pain rarely occur in the affected area. High fever is not common [17]. After BCG vaccination, first ulceration and then granulation tissue develops in the vaccination area, and when the bacillus reaches the regional lymph nodes via the lymphogenous route, a primary complex occurs. If the lymph node barrier is crossed, spread to organs such as the liver, lung, spleen, and kidney may occur via hematogenous spread. Sternal osteomyelitis may also occur as a result of hematogenous spread [6].

Talbot et al. classified BCG vaccine complications into four groups: (1) regional disease, (2) extra-regionally localized disease, (3) disseminated disease, and (4) other BCG syndromes. Recently, Hesseling et al. rearranged this classification as (1) local disease, (2) regional disease, (3) distant disease, (4) disseminated disease, and (5) other BCG syndromes [6]. According to this renewed classification, our patient can be included in the extra-regionally localized disease or distant disease groups.

In BCG osteomyelitis, inflammatory markers, such as erythrocyte sedimentation rate and C-reactive protein, mildly to moderately increase. Imaging methods are helpful in diagnosis, but it cannot be said that there are pathognomonic changes. When chest radiography is normal or tuberculosis complications are suspected, thoracic CT is a frequently used imaging method in children. In a recent review on children with culture-confirmed tuberculosis pneumonia, it was shown that 20% of patients had a normal chest radiograph and CT was required to demonstrate lung involvement. CT findings include soft tissue swelling, cortical infarction, and well-circumscribed osteolytic lesions in the metaphysis. These findings can also be seen in pyogenic, tuberculosis, syphilis, and fungal osteomyelitis [18]. In our patient, CT scanning helped distinguish the extent of destruction of the sternum and the involvement of the adjacent subcutaneous tissue mass.

In a study conducted in Japan, a retrospective review of clinical features and radiographic findings was performed to describe the pathology, frequency, and consequences of chest wall lesions in children, and in the differential diagnosis, lesions such as sternal deformities, bifidosis, sternal tuberculosis, osteochondroma, chondrosarcoma, Ewing sarcoma, and primitive neuroectodermal tumor were defined [19]. For a definitive diagnosis of BCG osteomyelitis, the BCG strain must be identified in the abscess or biopsy materials [20]. Although multiplex PCR examination has recently become a rapid and highly reliable diagnostic method, in our patient, the tuberculosis PCR results obtained from fasting gastric juice and tissue were negative. The diagnosis was made histopathologically.

Most cases of BCG osteomyelitis are treated with oral antituberculous therapy or surgical debridement. There is no established effective treatment for BCG osteomyelitis. A 6-12-month treatment with a regimen of isoniazid and rifampicin is often recommended. Ethambutol is avoided due to its recently diagnosed optic neuritis side effect. Pyrazinamide (PZA) is not included in the treatment because members of the Mycobacterium tuberculosis complex other than Mycobacterium bovis are sensitive to PZA, one of the main anti-tuberculosis drugs, while M. bovis isolates show natural resistance to the drug [14]. Our patient's organism was also sensitive to isoniazid and rifampicin, which are the isolated Mycobacterium bovis strains.

If the treatment is started early, the prognosis is extremely good, but if it is delayed, inflammation can spread from long bones to the joints, restricting joint movement and causing deterioration in bone growth. Although congenital immunodeficiency conditions such as interferon-γ receptor deficiency are seen together with multifocal BCG osteomyelitis, it was determined that most patients with osteomyelitis have a single lesion and do not have immune dysfunction [13]. No pathology was detected in the immunological examinations of our patient. As a summary, in this paper, a rare form of infective osteomyelitis, considered a complication of the BCG vaccination administered nine months ago, was presented. Our patient was diagnosed quickly, the treatment process started without wasting time, no complications developed during the treatment, and the patient recovered without sequelae. In countries where the BCG vaccine is administered regularly, if an atypical abscess due to bone lesions is detected in patients between 6 and 24 months after vaccination, BCG osteomyelitis should be considered, although rare.

## Conclusions

In conclusion, BCG osteomyelitis and osteitis require prompt diagnosis and appropriate intervention to prevent further major complications. In cases of young children who present with mild pain and swelling of the soft tissues alongside unremarkable laboratory tests, clinicians should be alert to the possibility of skeletal TB. In countries where the BCG vaccine is administered regularly, if an atypical abscess due to bone lesions is detected in patients between 6 and 24 months after vaccination, BCG osteomyelitis should be considered, although rare. The BCG vaccine remains useful, but physicians should be aware that this vaccine can also cause osteomyelitis and initiate prompt investigation and treatment if there are suspicious symptoms or findings. Examination, appropriate debridement, and the administration of antituberculous agents led to the complete recovery of our patient, without short-term sequelae.
